# The Luteal Phase after GnRHa Trigger-Understanding An Enigma

**Published:** 2014-11-01

**Authors:** Kathrine Leth-Moller, Sandra Hammer Jagd, Peter Humaidan

**Affiliations:** 1 Faculty of Medical Science, University of Southern Denmark, Odense, Denmark; 2 The Fertility Clinic, Skive Regional Hospital, Skive, Denmark; 3 Faculty of Health, Aarhus University, Aarhus, Denmark

**Keywords:** HCG, GnRHa, Luteal Phase, IVF

## Abstract

The luteal phase of all stimulated *in vitro* fertilization/intra-cytoplasmic sperm injection (IVF/ICSI) cycles is disrupted, which makes luteal phase support (LPS) mandatory. The cause of the disruption is thought to be the multifollicular development achieved during ovarian stimulation which results in supraphysiological concentrations of steroids se- creted by a high number of corpora lutea during the early luteal phase. This will directly inhibit luteinizing hormone (LH) secretion by the pituitary via negative feedback at the level of the hypothalamic-pituitary axis, leading to a luteal phase defect. With the intro- duction of the gonadotropin-releasing hormone (GnRH) antagonist protocol, it became feasible to trigger final oocyte maturation and ovulation with a single bolus of GnRH agonist (GnRHa) as an alternative to human chorionic gonadotropin (hCG). GnRHa trig- gering presents several advantages, including the reduction in or even elimination of ovarian hyperstimulation syndrome. Despite the potential advantages of GnRHa trig- gering, previous randomized controlled trials reported a poor clinical outcome with high rates of early pregnancy losses, despite supplementation with a standard LPS in the form of progesterone and estradiol. Following these disappointing results, several studies now report a luteal phase rescue after modifications of the LPS, resulting in a reproductive outcome comparable to that seen after hCG triggering. We herein review luteal phase dif- ferences between the natural cycle, hCG trigger and GnRHa trigger and present the most recent data on handling the luteal phase after GnRHa triggering.

## Introduction

### The luteal phase of the natural cycle

In the natural cycle ovulation is induced by a mid-cycle surge of luteinizing hormone (LH) and follicle-stimulating hormone (FSH) from the pituitary elicited by a rise in the late follicular phase level of estradiol and progesterone. Ovulation marks the transition from follicular phase to luteal phase, characterized by the formation of a corpus luteum which releases steroid hormones, including progesterone and estradiol. Importantly, the steroid production is totally dependent on the pulsatile secretion of LH by the pituitary (1, 2).

Apart from securing the function of the corpus luteum, LH plays a crucial role during the luteal phase by up-regulating growth factors like vascular endothelial growth factor A (VEGFA) and fibroblast growth factor 2 (FGF2). In addition, cytokines are up-regulated and extragonadal LHreceptors are activated in the endometrium. All these factors are thought to enhance and support implantation and early neo-vascularization (1, 3).

The role of the previously described mid-cycle FSH surge during the natural cycle is not fully understood; however, FSH seems to promote oocyte nuclear maturation, i.e. resumption of meiosis and cumulus expansion. Furthermore, FSH has been shown to induce LH receptor formation in the luteinizing granulosa cells, thus optimizing the function of the corpus luteum (1, 3).

Following implantation, the embryo gradually begins to secrete human chorionic gonadotropin (hCG) into circulation, structurally and biochemically similar to LH (3). The function of the corpus luteum requires a consistent hCG secretion until the luteo-placental shift around the 7th week of gestation, after which the placenta will be responsible for the continued secretion of steroids. The progesterone produced by the corpus luteum induces secretory transformation of the uterine glands, increases the endometrial vascularization and stabilizes the endometrial receptivity in preparation for embryo implantation. In addition, progesterone promotes uterine musculature quiescence thought to prevent uterine contractions, which could lead to expulsion of the embryo from the uterine cavity (2).

Apart from progesterone, the corpus luteum produces other steroid hormones, including estradiol. Estradiol has a modulatory effect on the secretory endometrial progesterone receptor concentration and may serve to replenish and maintain a sufficient level of endometrial receptors to secure an adequate response to progesterone (4).

### The luteal phase of the stimulated IVF/ICSI cycle

Controlled ovarian stimulation (COS) is a key component of modern IVF treatment, as the availability of multiple oocytes for fertilization increases the chance of pregnancy (5).

In the stimulated *in vitro* fertilization/intra-cytoplasmic sperm injection (IVF/ICSI) cycle, the luteal phase is physiologically abnormal. The most plausible reason for this is the multifollicular development achieved during the follicular phase, leading to a high number of corpora lutea. The collective steroid production of the corpora lutea will result in supraphysiological concentrations of progesterone and estradiol which directly inhibit the LH secretion by the pituitary via negative feedback actions at the level of the hypothalamic-pituitary-gonadal-axis (3). If un-supplemented, this will result in corpus luteum demise and early pregnancy loss (2). Therefore, to ensure the reproductive outcome of an assisted reproductive treatment (ART) cycle it is crucial to correct the luteal phase. This can be achieved either by increasing the early luteal LH activity or by supplementing with steroid hormones (1) until the circulating hCG produced by the implanting embryo is sufficiently high to secure the function of the corpus luteum.

### The luteal phase after hCG triggering

HCG has been the golden standard for ovulation induction for decades, functioning as a surrogate for the mid-cycle LH surge. HCG binds to and activates the same receptor as LH, the LH/hCG receptor (1), and, thus, by injecting a single bolus of hCG it is possible to trigger final oocyte maturation and ovulation.

After triggering of ovulation, it is necessary to maintain the function of the corpus luteum in order to secure a good reproductive outcome. HCG has a significantly longer half-life than that of endogenous LH and the bolus injected to trigger ovulation can support the corpus luteum for 7-10 days. After this period hCG is cleared from the circulation, and the corpus luteum is now totally dependent on the endogenous LH production and the hCG produced by the implanting embryo. However, during the early/mid luteal phase the hCG secretion from the embryo to the maternal serum is limited due to an absence of direct vascular communication (3). Moreover, the endogenous LH production is reduced by the supraphysiological concentrations of steroid hormone seen after COS. Thus, the result is a low LH activity leading to a decreased corpus luteum function during the early/mid luteal phase, necessitating luteal phase support (LPS) (1).

The best LPS strategy has yet to be defined, however progesterone administered either vaginally or intramuscularly is the first choice of treatment and is generally used for at least 15 days. Currently, the literature does not find any evidence for adding estradiol (2).

Regarding hCG triggering, the downside is that the trigger agent is very closely connected to the ovarian hyperstimulation syndrome (OHSS). This life threatening condition, characterized by massive enlargement of the ovaries and an increased vascular permeability, among others, is a iatrogenic complication following COS (5, 6). The main cause of OHSS is a combination of ovarian hyperstimulation with exogenous gonadotrophins and the use of hCG for final ovulation induction (6). Thus, hCG causes a sustained luteotropic activity due to its prolonged circulating half-life (1, 7). Furthermore, even low doses of hCG during the luteal phase have been shown to affect the expression of vascular mediators, thereby increasing the vascular permeability (1).

The best strategy to prevent OHSS has previously been to indentify high-risk patients before ovarian stimulation, followed by the use of an appropriate COS protocol (8).

### The luteal phase after GnRHa triggering

With the introduction of the GnRH antagonist protocol for the prevention of a premature LH surge, it became possible to trigger ovulation with GnRHa. The GnRH antagonist occupies the GnRH receptor without causing down-regulation, and by injecting a single bolus of GnRHa, the antagonist is displaced from the receptor. This activates the receptor, inducing a flare-up of gonadotrophins (LH and FSH), which effectively stimulate the final oocyte maturation and ovulation. However, important differences exist regarding the profile and duration of the LH surge after triggering with GnRHa compared to that of the natural cycle. In the natural cycle, the LH surge is characterized by three phases with a total duration of ~48 hours. After GnRHa triggering, the surge consist of two phases, only, with a duration of ~24-36 hours leading to a significantly reduced amount of LH released (1).

Apart from an LH surge, GnRHa triggering also induces an initial secretion of FSH resembling that of the natural cycle. This more natural surge of gonadotrophins after triggering with GnRHa may explain why some authors reported retrieval of an increased amount of mature oocytes compared to hCG triggering (1, 3).

The induced surge of gondotrophins results in an initial rise in the levels of progesterone and estradiol followed by a decrease during the next 24 hours prior to oocyte pick-up (OPU). Subsequently a second rise in the level of progesterone takes place as ovarian steroidogenesis shifts from follicular to luteal phase. In contrast, the estradiol level continues to fall (1).

After GnRHa trigger the circulating levels of progesterone and estradiol are significantly lower throughout the luteal phase as compared to those obtained after hCG triggering due to the shorter half-life of LH (~60 minutes) compared to that of hCG (>24 hours) (1).

The important clinical advantage of GnRHa triggering, however, is the reported significant reduction in or even elimination of OHSS (1, 3) caused by the shorter half-life of the endogenous LH surge compared with the continuous LH/hCG receptor stimulation after hCG triggering (1, 7).

As previously mentioned, the luteal phase after COS is defect due to supraphysiological steroid hormone concentrations inhibiting the LH secretion via negative feedback at the level of the hypothalamic-pituitary-gonadal-axis. As seen above the LH activity will be further compromised after GnRHa triggering due to the shorter duration of the endogenous induced LH surge and a potential weaker activation of the LH/hCG receptor. The result of this is a significant reduction in LH activity throughout the early/mid luteal phase leading to premature luteolysis and implantation failure (1).

In contrast, after hCG triggering, the luteal actions of LH will be covered by the bolus of hCG injected and then gradually by the hCG produced by the implanting embryo. Thus, supplementation with progesterone is sufficient to secure the reproductive outcome. However, after GnRHa triggering the lack of endogenous LH activity necessitates a modification of the standard luteal phase supplementation currently used after hCG triggering (1, 3).

### Modified luteal phase support after GnRHa triggering

The initial randomized controlled trials (RCTs) reported a poor clinical outcome with an extremely high early pregnancy loss rate (EPL) when GnRHa was used to trigger final ovulation, despite the use of standard LPS with vaginal progesterone and oral estrogen (9-11). The most plausible reason for the poor results was a suboptimal LPS, and it was clear that the luteal phase after GnRHa triggering was significantly different from that seen after hCG triggering and a search for a more optimal LPS commenced.

### A bolus of hCG

After the first disappointing results, trials were performed to explore the possibility of correcting the luteal phase by injecting a small bolus of LH activity in the form of hCG. HCG in standard doses would increase the risk of OHSS, but by supplementing with a reduced dose, the treatment was thought to be safe, even for the OHSS high risk patient. Therefore, trials were conducted to explore the hypothesis that a small hCG bolus could rescue the luteal phase without increasing the risk of OHSS (5, 9, 12-15).

Humaidan et al. (9) conducted a RCT, randomizing 302 normoovulatory women undergoing IVF/ICSI to ovulation induction with either hCG or GnRHa. The GnRHa group was supplemented with 1500 IU hCG 35 hours after triggering besides a standard progesterone and estradiol support. The study reported delivery rates (DR) and early pregnancy loss rates (EPL) comparable to those of hCG trigger. In the group of women triggered with hCG, the OHSS incidence was 2% as compared to no cases after GnRHa triggering. The trial included normo-ovulatory women, only, and it was unknown whether the protocol would be safe for the OHSS high risk patient. However, the authors assumed it to be safe as more than one third of the patients in each group had more than 14 follicles .11 mm on the day of triggering-a level previously set to predict the occurrence of OHSS.

Following this study, the question to ask was: does GnRHa triggering followed by a bolus of 1500 IU hCG in a group of patients at risk of OHSS reduce the OHSS incidence compared with hCG trigger? This question was explored in a more recent study by Humaidan et al. (16), including 390 women undergoing IVF/ICSI. The study consisted of two RCTs, one study, randomizing patients at risk of OHSS and another, randomizing patients at low risk of OHSS. The ovarian response on the day of final oocyte maturation was used to create two risk groups with a cut-off level of >14 follicles .11 mm. The group at risk of OHSS had final oocyte maturation with either a bolus of GnRHa followed by a single bolus of 1500 IU hCG (n=60) or 5000 IU hCG (n=58). Similarly, women at low risk of OHSS were allocated to receive either a bolus of GnRHa followed by a total of two boluses of 1500 IU hCG, on the day of OPU and OPU+5 (n=125) or 5000 IU hCG (n=141). For luteal phase support all patients received a standard progesterone and estradiol supplementation.

No OHSS cases were seen in the group at risk of OHSS after GnRHa triggering despite supplementation with 1500 IU hCG, compared to an incidence of 3.4% in the group at risk of OHSS triggered with hCG. In contrast, two late-onset moderate OHSS cases were seen in the OHSS low-risk group triggered with GnRHa followed by two boluses of 1500 IU hCG, versus no cases of OHSS after hCG triggering. The authors concluded that future trials should focus on the minimal hCG activity needed for LPS in the low risk group to secure the reproductive outcome without increasing the risk of OHSS. The safety of the LPS protocol was previously tested among 12 hyper responders (15). Patients had a mean of 22 oocytes and all had embryo transfer (ET), which resulted in a live birth rate (LBR) of 50%. One moderate late-onset OHSS case occurred; however, the patient did not require hospitalization.

Using the same protocol, Radesic et al. (5) retrospectively explored its safety and efficiency in 71 OHSS high risk patients. The authors reported that the use of a GnRHa trigger in combination with 1500 IU hCG on the day of OPU resulted in a high ongoing pregnancy rate (OPR) of 52% without increasing the risk of severe OHSS. Despite the average patient producing a mean of 17 oocytes, only one patient (1.4%) was hospitalized due to OHSS in this high-risk group. All patients were supplemented with daily progesterone and estradiol.

These results were further corroborated by the conclusions of a recent international multicentre retrospective study. In this study Iliodromiti et al. (17) included 275 women at high risk of OHSS. The study reported an overall CPR of 42% and two cases of severe OHSS, only, (0.72%). The authors concluded that in women undergoing ovarian stimulation and who develop an excessive ovarian response, the use of a GnRH agonist trigger combined with a bolus of 1500 IU hCG at the time of oocyte retrieval provides an opportunity to proceed with fresh embryo transfer.

In another effort to find the optimal dose of hCG necessary during the luteal phase after GnRHa triggering, Castillo et al. (13) reported the outcomes of a retrospective study including 192 patients undergoing IVF/ ICSI from 2002-2006. Throughout the study period, the treatment protocol for luteal hCG administration changed, and the patients were grouped based on the dose received: group A (n=44) received 1000 IU, group B (n=115) received 500 IU and group (n=33) received 250 IU hCG i.m. A total of three fixed doses of hCG were administered starting on the day of OPU and every third day along with daily progesterone supplementation. Regarding the mean number of embryos transferred, there was a significant difference between the groups due to an actual trend of transferring fewer embryos. Despite these differences, the groups were comparable as regards EPL and clinical pregnancy rate (CPR). There was a clear trend of fewer cases of OHSS when using lower doses of hCG and the authors concluded that three doses of 1000 IU of hCG was unadvisable. The vast majority of the severe OHSS cases were late-onset (6/7), and 4/6 were related to multiple pregnancies. The results show a distinct relationship between the risk of OHSS and multiple pregnancies-single embryo transfer is therefore highly recommended in all patients at risk of OHSS development, regardless of trigger mode.

In another retrospective analysis, Shapiro et al. (12) reported a high ongoing pregnancy rate and no OHSS cases among 182 OHSS high-risk patients, using a so-called "dual-trigger". Patients received ovulation induction with a bolus of GnRHa as well as an average dose of 1428 IU of hCG, followed by LPS with progesterone and estradiol. Although retrospective, the results seem to indicate that dual-triggering can correct the luteal phase without causing OHSS among OHSS high risk patients.

Finally, Kol et al. (14) explored for the first time, the use of a LPS protocol without exogenous progesterone and estradiol after GnRHa triggering. The study included 15 normal responder patients with .12 follicles, who were supplemented with two boluses of 1500 IU of hCG, only, during the luteal phase. The boluses were administered on the day of OPU and OPU+4. The study reported an OPR of 47% and no OHSS development in any of the patients. These results seem promising as they might introduce the future exogenous progesterone free LPS for the normo-reponder IVF patient triggered with GnRHa.

In conclusion, supplementation with hCG rescues the luteal phase after ovulation induction with GnRHa, resulting in reproductive outcomes similar to that of hCG. However, it still needs to be determined whether "dual-trigger", a single bolus of hCG or repeated low-doses of hCG is the best option. Regardless of the chosen protocol, it is crucial to individualize the luteal phase treatment with hCG according to the ovarian response to stimulation in an effort to reduce the risk of OHSS.

### Recombinant LH

An alternative way of increasing the LH activity during the insufficient luteal phase after GnRHa triggering would be to administer repeated doses of recombinant LH.

This concept was explored in a proof-of-concept study performed by Papanikolaou et al. (18). The study included 35 normal responder patients randomized to receive ovulation triggering with either GnRHa or hCG. All patients received elective single embryo transfer (SET) after having undergone the same stimulation protocol. In the GnRHa group the luteal phase was supported with six alternate doses of 300 IU rLH, starting on the day of OPU and repeated every other day in addition to 600 mg daily of progesterone, administered vaginally. The study reported DR and EPL rates comparable to those of hCG trigger and no cases of OHSS were seen in either group.

The authors in this small group of normo-responder patients concluded that rLH effectively secures a good reproductive outcome after triggering with GnRHa without any OHSS development. The study was the first to assess the concept of applying repeated doses of rLH as LPS to overcome the luteal phase insufficiency after GnRHa triggering and the results seem promising. However, larger RCTs are necessary to draw conclusions about the safety and efficacy of this protocol. Furthermore, an obvious limiting factor for the use of rLH for LPS is the high cost of this preparation.

### Intensive progesterone and estradiol support

As the standard LPS regimens turned out to be insufficient after GnRHa trigger (10, 11), US based research groups explored the use of a LPS protocol, consisting of progesterone and estradiol, only (6, 8, 12, 19).

The first report was by Engmann et al. (8) who randomized a total of 65 PCOS patients undergoing IVF treatment. The patients were allocated to an ovarian stimulation protocol consisting of either GnRHa trigger after co-treatment with GnRH antagonist or hCG trigger after dual pituitary suppression, using a long GnRHa down-regulation protocol. The luteal phase supplementation in the GnRHa trigger group consisted of intensive support with progesterone and estradiol. Luteal serum levels were closely monitored and the administration of progesterone and estradiol was adjusted to maintain serum levels of >20 ng / ml and >200 pg/ml, respectively.

This protocol resulted in DR and EPL rates comparable to those of hCG triggering. Furthermore, the study reported a total elimination of OHSS after GnRHa triggering despite the fact that PCOS patients were included, many of which were at high-risk of developing OHSS after ovarian stimulation.

An important question that needs to be explored is whether this protocol applies to normo-gonadotrophic patients. LH levels are significantly higher in PCOS patients during the follicular and luteal phases, due to a higher frequency and amplitude of the LH pulse. Further, in PCOS patients the hypothalamus has a reduced sensitivity to negative feedback from the ovarian steroid hormone concentrations, in particular progesterone (1). This leaves PCOS patients with a significantly higher LH level during the luteal phase as compared to the normo-gonadotrophic patient.

In accordance with the results from Engmann et al. (8) and Shapiro et al. (12) reported good pregnancy outcomes and no cases of OHSS after the use of intensive LPS with progesterone and estradiol among 24 high responders. In their study, the luteal phase was also closely monitored to maintain levels of progesterone and estradiol of ≥15 ng/ml and ≥200 pg/ml, respectively.

Although the results seem promising in terms of the reproductive outcome and the total elimination of OHSS among OHSS high-risk patients, the study by Shapiro et al. (12) is obviously limited by its design and small study population and the results need to be confirmed in future larger RCTs.

Moreover, there are some potential biases in the study by Engmann et al. (8). Thus, a long GnRHa protocol was compared with a GnRH-antagonist/GnRHa protocol and the individualized LPS was only administered to the GnRHa group.

Importantly, the abovementioned encouraging results are contrasted by others. In a previous study performed by Babayof et al. (6) 28 PCO patients considered at high-risk of developing OHSS were randomized to receive either GnRHa or hCG triggering. Patients received intensive luteal support with 50 mg/day i.m. progesterone and the dose was doubled at serum levels of <12.5 ng/ml. Moreover, 4 mg/day of oral estradiol was given at serum levels <200 pmol/l. Despite the intensive LPS the reproductive outcome was disappointingly low with an OPR of 6% and an EPL of 80% in the group of patients receiving GnRHa for triggering.

These findings are further supported by Orvieto (19) who reported a low reproductive outcome in 67 OHSS high-risk patients despite the fact that they had a LPS protocol similar to the one suggested by Engmann et al. (8). Thus, there is clearly a need for RCTs to clarify the efficacy of the intensive progesterone and estradiol protocol among high-risk as well as low-risk patients.

### Segmentation strategy

An alternative approach to encounter the luteal phase insufficiency seen after GnRHa triggering is to segmentate the IVF cycle, i.e. to stimulate in one cycle, trigger with GnRHa and transfer in subsequent frozen-thaw cycles. This seems to be a very safe approach for patients at risk of OHSS and recent trials suggest similar pregnancy rates between fresh and frozen-thawed embryos (20-22).

The concept was recently explored in a RCT by Shapiro et al. including 177 patients (20). A blastocyst transfer was performed in 103 patients. In the group randomized to fresh embryo transfer the final oocyte maturation was induced with either hCG alone or, using dual-triggering. The group receiving frozen-thawed embryos had a significantly higher reproductive outcome per transfer compared to the fresh transfer group. The authors concluded that the difference in outcomes probably was due to superior endometrial receptivity in the freeze all group.

To extend the indication, Shapiro et al. (21) conducted a similar study in high responder patients who were randomized to receive either fresh or frozen-thaw transfer. In both groups final oocyte maturation was induced with the use of a dual-trigger. The CPR was 80 vs. 65%, in favour of frozen-thaw ET despite a superior embryo quality in the fresh group.

In summary, a freeze-all strategy further reduces the risk of OHSS and may be the best current option for patients with a very high risk of OHSS (22). However, there is a need for future trials to justify the use of oocyte/embryo cryopreservation as a routine approach. Importantly, this approach demands access to optimal cryopreservation programs (3).

## Conclusion

Many recent publications indicate that the time has come for a paradigm shift in the triggering policy of ART. HCG has been the gold standard for ovulation induction, however, after the introduction of the GnRH antagonist protocol for the prevention of a premature LH rise, triggering of final oocyte maturation and ovulation with a single bolus of GnRHa is definitely an alternative.

GnRHa triggering possesses important advantages over hCG triggering, mainly in terms of a significant reduction in-if not total elimination of OHSS. Following the initial disappointing clinical reports several subsequent studies implemented a modified luteal support in terms of supplementation with either LH activity or luteal steroids. Using the modified LPS, the reproductive outcome increased remarkably and is now comparable to that seen after hCG triggering. Although the modified LPS has had a significant positive effect on the reproductive outcome after GnRHa triggering without increasing the risk of OHSS, the most optimal LPS still has to be investigated.

Until the optimal luteal supplementation protocol has been defined an alternative option in patients with an extreme ovarian response or with a significant comorbidity is a freeze all strategy and transfer in a subsequent natural or stimulated cycle.
